# Hyaluronic acid in Dentoalveolar regeneration: Biological rationale and clinical applications

**DOI:** 10.1016/j.jobcr.2024.02.010

**Published:** 2024-03-12

**Authors:** Alaa Mansour, Anirudh Balakrishna Acharya, Charles Alliot, Nael Eid, Zahi Badran, Yousef Kareem, Betul Rahman

**Affiliations:** a Periodontology Unit, Department of Preventive and Restorative Dentistry, College of Dentistry, Sharjah University, United Arab Emirates; b Department of Periodontology, Faculty of Dental Surgery, University of Nantes, Nantes, France; c Prosthodontics Unit, Department of Preventive and Restorative Dentistry, College of Dentistry, Sharjah University, United Arab Emirates; d College of Dentistry, King Saud University, Riyadh, Saudi Arabia

**Keywords:** Hyaluronic acid, Biological effect, Periodontal regeneration

## Abstract

**Background:**

Hyaluronic acid (HA) is found in different locations in the periodontium, including mineralized tissues (i.e., cementum and alveolar bone) and non-mineralized tissues (i.e., gingiva and periodontal ligament). In addition, it seems to play an essential part in regulating the underlying mechanisms involved in tissue inflammatory reactions and wound healing. HA has the potential to regulate periodontal tissue regeneration and treat periodontal disease.

**Aim:**

The current review of the literature was conducted to assess how HA plays its part in periodontal therapy and examine the contemporary literature's viewpoint on its use in periodontal regeneration.

**Conclusion:**

HA has a multifunctional character in periodontal regeneration, and healing and appears to provide promising outcomes in different periodontal regenerative applications.

## Graphical abstract

**Figure:**
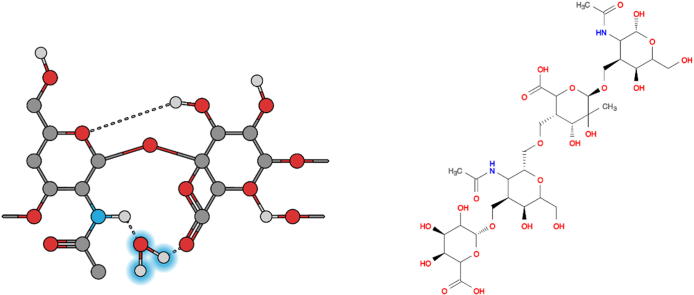

## 1 Introduction

Periodontitis is considered a chronic inflammatory disease initiated due to bacterial and host immune response interactions.^1^ As a result, tissue destruction occurs with the occurrence of periodontal defects which necessitates therapy that should be focused, whenever possible, on the regenerative capacity of the damaged tissues.^2^ The principal objective of the regeneration process is to re-establish the original architecture of the periodontium, which depends not only on the differentiating and proliferative potential of the existing cells into other cells to re-establish the damage but also the requirement of providing a suitable environment that supports and stimulates their activities. The concept of guided tissue regeneration has been introduced by Melcher which includes the use of barrier membranes to guide the biological process of periodontal wound healing by giving preference to particular cells having the capacity to regenerate the desired type of tissue (i.e., periodontal ligament cells).^3^ Different techniques can be applied for periodontal regeneration such as papilla preservation flap^4^ and minimally invasive surgical approach (i.e., Modified and simplified papilla flap).^5^ Additionally, multiple biomaterials (e.g. bone grafts, collagen membranes, enamel matrix derivatives) have been proposed for periodontal regeneration. Despite the successful outcomes obtained with the aforementioned modalities, their relatively high cost is still a challenging factor that may affect the treatment plan approval by most of the affected individuals.^6^^,^^7^ This has driven tissue engineering researchers to construct scaffolds that provide sufficient mechanical and biological support for the functional activity of cells involved in periodontal regeneration such as fibroblasts, cementoblasts, and osteoblasts. In this regard, hyaluronic acid (HA) has been suggested as an alternative biomaterial with promising regenerative outcomes and distinctive biological and physicochemical functions, which can be cost-effective.^8^

In dentistry, HA has been proposed to be a suitable material for periodontal wound healing, gingival recession treatment, and the regeneration of intra-bony periodontal defects with promising results in improving the clinical attachment level and reducing probing depth (PD). A possible explanation for these outcomes is the positive effect of HA on enhancing the viability and proliferation of periodontal ligament fibroblasts and osteoblast differentiation that results in a significant increase in collagen production and new bone formation.^9^^,^^10^

It is of interest to know about the various applications of HA in periodontal regeneration, and therefore, in this narrative review, we will discuss the potential application of HA in periodontal and peri-implant regenerative procedures.

## 2 History

In 1934, Karl Meyer and John Palmer discovered HA by isolating a substance from the vitreous fluid of the eye of a cow, which was later given the name “Hyaluronic Acid”.^11^ It is a foremost constituent of the extracellular matrix (ECM) in several tissues as this non-sulfated glycosaminoglycan is synthesized by different types of cells, including those of periodontium such as keratinocytes and fibroblasts, in the gingival tissue, periodontal ligament, cementoblasts, and osteoblasts, and therefore, is a structural component found in different parts of the periodontium.^12^

## 3 Structure and biosynthesis

HA occurs naturally as a non-sulfated glycosaminoglycan (GAG) that consisting of repeating disaccharide units in a linear chain that is linked by β-1,4-glycosidic bonds. Each disaccharide units contains N-acetyl-d-glucosamine and d-glucuronic acid connected by β 1,3-glycosidic bonds.^13^ Unlike other GAGs (such as dermatan sulfate, heparin, heparin sulfate, keratin sulfate, and chondroitin sulfate), the plasma membrane mainly produces HA through a group of hyaluronan synthase isoenzymes, where a membrane-bound protein synthesizes HA via the transport of activated monosaccharides to GAG chains, and releasing uridine diphosphate that is directly secreted into extracellular spaces.^14^ A high concentration of HA could be found in the umbilical cord, epidermis, and synovial fluid, whereas, the lowest concentration is present in the blood serum. HA synthase enzymes present in multiple cell types (e.g., fibroblasts, periodontal ligament cells, cementoblasts, osteoblasts) synthesize HA in the periodontal tissues. Large quantities of HA in periodontal tissues are found in the connective tissue of the gingiva, and periodontal ligament compared with cementum and alveolar bone where low concentrations are present.^11^ The tissue turnover of HA content may occur either because of the draining lymphatics into the bloodstream or by regional metabolism, which influences the half-life of HA ranging between 12 h and 3 days.^15^ HA's enzymatic degradation occurs through the involvement of certain enzymes including hyaluronidase (hyase), β-N-acetyl-hexosaminidase, and b-d-glucuronidase. Hyaluronidase acts by cleaving HA that has a high molecular weight (MW) into smaller oligosaccharides, while the oligosaccharide can be further degraded into fragments by removing non-reducing terminal sugars by β-N-acetylhexosaminidase and β-d-glucuronidase.^16^

## 4 Biological effects

### 4.1 HA in the wound-healing process

Following injury, wound healing occurs through well-regulated steps including inflammation, granulation tissue formation, reepithelization, and remodeling. HA is known to be an essential part of the wound-healing process, which is probably related to its structural role as an integral part of the extracellular matrix and the ability to impact cellular behavior.^17^

During the inflammation phase, a provisional matrix rich in HA is formed in the wound area that results in interesting biological effects such as maintaining the structural integrity of ECM, influencing cellular adhesion, proliferation, and differentiation, and impacting the level of gene expression.^18^ The MW of HA largely is responsible for determining its biological effects. Molecules with an MW between 0.4 and 4.0 kDa can induce inflammation and increase the expression of pro-inflammatory cytokines such as IL-1β, IL-8, and TNF-α. Whereas, high MW HA (>500 kDa) possesses an anti-angiogenic effect and immunosuppressing function.^19^ Moreover, the hydrophilic nature of this matrix creates an environment favoring cell migration which is a fundamental step toward wound healing.

Similarly, the HA content in the granulation tissue matrix seems to regulate cell functions and survival thanks to its physicochemical properties and direct interaction with cells through a group of HA-specific cell receptors including CD44, RHAMM, and ICAM-.^20^ CD44 is considered the main cell surface receptor for hyaluronan, which is suggested as the inducer of several physiologic events such as cell migration and proliferation, as well as HA uptake and degradation.^21^ The expression of the RHAMM (Receptor for hyaluronan-mediated motility) could be detected on a wide variety of motile cells (e.g., fibroblasts) and it is mainly associated with cell migration.^22^ In addition, the binding of hyaluronan to ICAM-1 may affect its binding to other receptors such as the leukocyte integrins lymphocyte function associated-1 (LFA-1).^23^

HA-binding proteins, also known as hyaladherins, facilitate HA binding with other matrix components (i.e., proteins, cells), which helps matrix assembly and contributes to cell-matrix and cell–cell interactions.^24^ More importantly, HA oligosaccharides can stimulate angiogenesis required for tissue-forming cell proliferation and differentiation, and eventually leading to wound healing.^25^

In addition, HA exhibits anti-inflammatory properties by suppressing the production of prostaglandins and metalloproteinases in addition to the effective reactive oxygen species scavenging activity.^26^ Another study demonstrates the possible role of HA in affecting the process of fibrin clotting by decreasing the time required for its formation.^27^ Collectively, this could explain its important clinical role in wound healing and periodontal regeneration.

### 4.2 HA effects on cell activity

#### 4.2.1 Osteoblasts

HA assists in regulating the behaviors of osteogenesis-related cells through either indirect (i.e., physicochemical properties) or direct mechanisms (i.e., binding to cell surface receptors). For the latter, the CD44 receptor is highly expressed, and binding to HA regulates the behavior of osteogenic cells. While binding to RHAMM is associated with controlling cell motility. On the other hand, it has been demonstrated that HA with different molecular weights exhibits variable biological functions during the process of bone regeneration. HA molecules with high molecular weight (i.e., 900 and 2300 kDa) enhance mRNA expressions of osteogenic gene markers (i.e. RUNX-2, ALP, and OCN), however, other isoforms with low molecular weight (i.e. 60 kDa) could promote cell proliferation and differentiation.^28^^,^^29^

#### 4.2.2 Fibroblasts

Periodontal ligament cells (i.e., Fibroblasts) play a crucial role in periodontal wound healing due to their ability to differentiate into other cell types including bone cells and cementoblasts. Interestingly, HA could strongly affect their behavior through certain signaling molecules (i.e., Akt, Erk1/2, and p38). In vitro experiments indicate the potent effect of HA on increasing cell proliferation and migration capabilities and triggering the expression of COL3A1 and TGFB3 genes necessary for scar-free wound healing. Furthermore, HA has the ability to upregulate the genes encoding growth factors essential for healing such as PDGFB, FGF‐2, and EGF, and affect ECM remodeling activity by stimulating the expression of MMP genes (e.g., MMP1 and 8).^12^

#### 4.2.3 Cementoblasts

In vitro assessment shows significant improvement in cementoblasts behavior when treated with HA. In brief, exposure of cells to HA significantly improves their viability and ability to produce mineral-producing capacity which is evident in increased expression of mineralized tissue markers including COL-I, BSP, RunX2, ALP, and OCN.^30^

### 4.3 HA and alveolar bone regeneration

HA has several favorable characteristics that give it a promising role in alveolar bone regeneration. Evidence exists in the literature regarding this via various animal models and in vitro investigations. HA is water soluble, viscous, homeostatic, and hygroscopic.^31^ It is also non-immunogenic,^32^ bacteriostatic,^33^ and most importantly, biocompatible.^34^ HA stimulates fibroblast proliferation,^35^ collagen synthesis and deposition,^36^ affects osteogenic differentiation, and enhances osteoblast activity.^37^

HA-based hydrogel scaffolds combined with other bioactive agents have shown the potential to stimulate alveolar bone regeneration.^38^ For instance, the bone mineral density and the total amount of bone regeneration by using bone morphogenetic protein (BMP)-2 and vascular endothelial growth factor (VEGF) incorporated with an HA-based hydrogel were higher compared with controls.^39^ Collagenase encapsulated with a hydroxyapatite-HA composite,^40^ HA microspheres loaded with platelet lysates,^41^ have been examined in alveolar bone regeneration with satisfactory effects. Owing to the challenges of in vivo maintenance of growth factors and osteogenic factors in defect sites, scaffolds having HA are potentially useful in alveolar bone regeneration outcomes.^42^ Ridge preservation has also been reported to be better with the use of HA in conjunction with other biomaterials such as absorbable collagen sponge and collagen membrane.^43^ HA (1%) combined with an absorbable collagen sponge was used to treat infected extraction sockets revealing HA promotes bone formation and osteocalcin expression.^44^ Overall, these findings illustrate that HA-based scaffolds provide a platform with promising outcomes for bone engineering applications and regenerative medicine. However, its poor mechanical properties and susceptibility to degradation in vivo make its use in bone regeneration questionable. One important criterion of bone regeneration scaffolds is the capability to maintain their mechanical integrity after implantation and bear specific loads to provide the ultimate protection for cell differential and growth, and consequently tissue formation.^45^ Therefore, it is essential to scrutinize the application of HA in human alveolar bone regeneration. In this regard, several strategies have been suggested to overcome such a limitation.

An approach to enhance the stiffness of HA-based hydrogels is by adding reinforcing agents into the scaffold matrix. Nanoparticles, with their diameter ranging from 1 to 100 nm, proved to provide 3D scaffold with improved mechanical support, in terms of increased compressive and toughness, which is necessary to ensure the proper survival rate of cells. Examples of nanoparticles used include carbon nanotubes, graphene oxide, and hydroxyapatite nanoparticles, and nanofibers made of biodegradable polymers such as poly (lactic-co-glycolic acid) (PLGA) and polycaprolactone (PCL).^46^ Additionally, the addition of strong biomaterials such as bioceramics and synthetic polymers can also produce stiff HA scaffolds with a controlled degradation rate, thus providing stability of these scaffolds during wound healing for better bone regeneration outcomes.

### 4.4 HA and regeneration of periodontal defect

More than two decades ago, Engstrom et al.^47^ investigated the injectable HA for its anti-inflammatory and bone regenerative effects on maxillary and mandibular periodontal bone defects of similar characteristics by non-surgical and surgical interventions in 15 patients. The radiographic observations at different time intervals (12 months being the last) pointed to an increased bone height of 2.2% (0.5 mm) in the test group surgically treated with HA and a bioabsorbable membrane. They also noted that HA in contact with bone and soft tissue did not elicit an immune effect because of the gingival crevicular fluid (GCF) estimation of IgG, prostaglandin E2(PGE2), and C3 levels. A few years later, an autologous bone graft with esterified HA (a product containing an ester of HA with benzyl alcohol) was tested in the surgical correction of periodontal infrabony defects in nine subjects.^48^ The surgical procedure did not involve barrier membranes. This study observed no bacterial contamination in all the surgical sites, attributing it to the bacteriostatic property of HA. The results noted a mean clinical attachment gain of 2.6 mm at 9 months. The authors report ‘mild bone remodeling’ and excellent bone fill at 6 months radiographically; satisfactory bone fill at 24 months by clinical and radiographic evaluation. A randomized clinical trial on forty individuals was conducted to assess the two-wall infrabony periodontal defects by open flap debridement with, and without HA.^49^ Although this study did not record bone changes, clinical attachment level (CAL) gain and PD reduction were significantly improved in the HA-treated group at 2 years. Additionally, High MW HA as a scaffold contributed, not only to the regeneration of alveolar bone, but also the formation of periodontal ligament and cementum in furcation defects.^50^

In another investigation, improvements in clinical parameters such as CAL, PD, gingival recession (REC), and probing bone levels were reported by de Santana et al.^51^ following the application of HA hydrogel scaffolds enriched with fibroblast growth factor type 2 (fhFGF-2) in the surgical treatment of periodontal intrabony defects. This implies that rhFGF-2 may have enhanced the improvements in the clinical parameters, which may be supported by the suggestion that HAmay not have significant regenerative potential alone, but only when incorporated with osteogenic factors.^52^ At this juncture, it is interesting that 1% HA as a stand-alone applied to extraction sockets compared with controls (no treatment, i.e., natural healing) showes a better percentage of bone formation after 30-days post-extraction.^53^ The early accelerated bone formation may be attributed to the binding of HA to proteins of the early healing cascade (fibrin, fibrinogen, fibronectin, collagen).^54^

HA in conjunction with guided tissue regeneration (GTR) has been attempted to enhance periodontal bone regeneration. Sehdev et al.^55^ in their randomized parallel design study demonstrated improvement in radiographic defect depth of the infrabony defect test sites treated with HA and a biodegradable GTR membrane. A randomized, controlled, split-mouth, clinical trial^56^ tested HA gel (0.8%) as an adjunct to open flap debridement in the treatment of periodontal intrabony defects. This trial evaluated clinical and radiographic parameters. Cone beam computed tomography (CBCT) compared the defect fill, alveolar crest changes, and defect resolution at 12 months from baseline. The test group (0.8% HA + open flap debridement) had significantly better CAL gain, defect fill, and reduction in PD when compared with the control group (placebo + open flap debridement). A most recent systematic review and meta-analysis concluded that HA + open flap debridement in the treatment of periodontal intrabony defects was significantly better compared with open flap debridement alone in CAL and PD outcomes at 6 months and that HA in combination with bone substitutes did not show significant differences.^58^

In case of a periodontal surgery is planned, efforts should be directed to provide treatment with less time consumption and postoperative complications while providing the desired therapeutic outcomes. For this purpose, a minimally invasive periodontal treatment was introduced in periodontics by Harrel and Rees for periodontal regeneration to maintain the integrity of the papillae through raising a conservative flap to access the periodontal defect(s). This also helps improve tissue handling of soft and hard tissues and improve clot stability.^59^ Therefore, Papilla preservation flap^4^ and single-flap approach^60^ should be the treatment whenever periodontal regeneration is required. This concept has been further enforced by Cortellini and Tonetti for the treatment of isolated periodontal defects by applying either the simplified papilla preservation flap (SPPF) in case of narrow interdental spaces or the modified papilla preservation technique (MPPT) in case of large interdental spaces.^61^^,^^62^ Overall, minimally invasive surgery proved its efficacy in periodontal regeneration,^63^ and it could be assumed that the adjunctive application of HA may further enhance the outcomes. This has been evaluated in a randomized controlled trial by Pilloni et al.,^57^ which showed that a single flap approach for treating intrabony defects combined with HA provided significant CAL gain and reduction in PD. These outcomes were comparable to those obtained with a combination of the same flap technique and enamel matrix derivative.

Collectively, the aforementioned studies demonstrate the efficacy of using HA in periodontal therapy as it seems to accelerate wound healing and improve the treatment outcomes. Nevertheless, further research in this field is recommended to optimize the administration of HA in periodontal therapy concerning the type of defects, dose, and duration of biomaterial application.

### 4.5 HA and regeneration in extraction sockets, sinus augmentation, and oral implants

The conclusion of the aforementioned systematic review and meta-analysis is a point to ponder regarding alveolar bone regeneration in other scenarios because reports in the literature suggest a beneficial role of adding HA with bone substitutes, but with contradiction. For example, Kaya et al.^64^ concluded that at 6, and 12 months, HA + xenograft + collagen membrane did not have an advantage over xenograft + collagen membrane in the repair of peri-implant dehiscence-type bone defects for implant placement. Whereas Lorenz et al.^65^ reported accelerated bone formation when HA was combined with alloplastic materials and autogenous bone in extraction sockets. Husseini et al.^66^ showed mixing cross-linked HA with demineralized bovine bone material (DBBM) compared with DBBM alone limited bone resorption in alveolar ridge preservation before implant placement as evaluated by CBCT at 4 months post-extraction.

Dogan et al.^67^ added HA to collagenated heterologous bone graft in sinus augmentation and noted a significantly higher percentage of bone formation compared with collagenated heterologous bone graft alone. A couple of case series by Schwartz et al.,^68^ and by Stiller et al.^69^ also indicate the positive regenerative effect of HA application with different bone grafts in sinus augmentation. Adding HA to bone substitutes for enhanced bone regeneration seems to be useful.

Although non-human studies^70^^,^^71^ do not clarify the role of HA in osseointegration, its seems to be favorable. A crossover randomized clinical trial on 30 dental implant patients by Lupi et al.^72^ assessed 52 HA-coated titanium implants against 48 uncoated controls and reported no differences between the two in terms of wound healing, bone resorption, implant stability, and success. However, Garunov et al.^73^ in their study on 128 dental implant patients, the surgical sites treated with hydroxyapatite + tricalcium phosphate + HA proved to be more efficacious than hydroxyapatite + tricalcium phosphate and natural healing regarding peri-implant bone remodeling and implant stability after one year.

Generally, the use of HA alone, in combination with other bone substitutes, (or as a scaffold or a carrier) for bone regeneration is an encouraging option.^74^^,^^75^ But the variety of varied methodologies, heterogeneous data, and conclusions makes it challenging to establish HA as a convincing regenerative material. Based on the highest level of evidence^58^ in this review, HA is valuable in the treatment of periodontal bone defects. However, further research is essential to endorse HA as a bone regenerative material in different periodontal and oral implant-related settings.

### 4.6 HA and periodontal soft tissue regeneration

Gingival recession is a common periodontal problem caused due to a variety of etiological factors such as traumatic brushing, and a periodontal disease, which usually results in tooth sensitivity and increased risk of root caries. For these reasons, root coverage procedures are usually indicated to treat areas of localized or generalized soft-tissue recession, especially in the anterior teeth where esthetics is a major concern to the patient.^63^ Treatment options for such a condition include the use of Pedicle grafts (e.g., laterally displaced flap, coronally displaced flap), or free grafts including Subepithelial connective tissue grafts (SCTG) and Free gingival grafts (FGG), Despite of the reliability of such therapeutic modalities, however, in some circumstances, surgical intervention is not recommended (e.g., smoking, or questionable dentition). Harvesting a SCTG is usually accompanied by some drawbacks such as including donor site morbidity, time-consuming, limited tissue availability, risk for palatal sloughing, bleeding, and pain.^65^ Therefore, looking for an alternative treatment modality is recommended to overcome such limitations. As a result, the use of allografts (i.e., Alloderm)^66^ and guided tissue regeneration (GTR)^67^ has been suggested.

Recently, research has focused on HA given its interesting biological properties such as anti-inflammatory, bacteriostatic, and anti-edematous properties. Furthermore, it has been shown to enhance clot formation, induce angiogenesis, and promote cell differentiation, adhesion, and migration in the course of the formation of tissue and reparative processes.^68^ Thus, HA can be considered a material that is suitable for the healing and regeneration of the periodontium.^69^^,^^70^ Studies have illustrated the adjunctive use of HA gel for root coverage is a promising alternative treatment option that could clinically result in significant root coverage and increase the keratinized tissue volume**.**

Despite the promising outcomes obtained with CAF, there is no guaranteed result in the regeneration of the periodontal tissues including cementum and periodontal ligament. In this regard, HA has been proposed as a capable adjunct with CAF in root coverage therapy to improve the outcomes obtained with such a procedure including CAL gain, reduction in PD, and significant improvement in complete root coverage (CRC) and mean root coverage (MRC)72. In the clinical study by Pilloni et al., the use of HA as an adjunct in combination with CAF surgery results in a significant increase in CRC when treating Miller's class I and II defects (80%) as compared with CAF alone (33.3%), 18 months postoperatively.^76^ Kumar et al. indicated that the application of CAF/HA combination resulted in a significant gain in the CAL after 24 weeks post-operatively, and this treatment approach may be suggested in cases where more stable results are needed.^77^ Additionally, the combination of the Hyaloss matrix with bio-absorbable membrane-like polylactic/polyglycolic acid (PLA/PGA) produces a significant enhancement in root coverage (92.93%), in comparison to the SCTG (84%) alone.^78^ Furthermore, a preclinical trial showed that the application of HA with CAF resulted in significantly increased regeneration of the periodontium (i.e., periodontal ligament, cementum, and alveolar bone formation) compared to when treating only with CAF. This presents CAF/HA combination as a novel option for promoting the regeneration of the periodontal tissues in gingival recession defects.^79^ On the other hand, although comparable root coverage outcomes for multiple gingival recession are obtained with or without the addition of HA to a modified coronally advanced tunnel technique combined with SCTG, better soft texture could be obtained when HA is applied.^80^

Intriguingly, the evolution of minimally invasive periodontal therapy provides a safe and convenient therapeutic approach for periodontal soft tissue deficiencies. In this regard, injecting HA is considered a minimally invasive strategy to correct the gingival and papillary recession in the esthetic zone by stimulating the migration of fibroblasts and fibrogenesis, with a reasonable duration of outcomes stability.^81^

## 5 Discussion

HA has initially been used in dentistry as a topical treatment of oral lesions/ulcers or to enhance postoperative mucosal healing after oral surgical procedures.^82^ Owing to the moisturizing, anti-inflammatory, and regenerative properties,^83^ the dental applications of HA have increased making it a valuable choice for strengthening tissues, promoting healing, and enhancing esthetic outcomes in surgical procedures.^84^ In the last years, growing interest in the bone regenerative properties of HA has been given in the scientific literature.

HA can functionalize bone substitutes and thus improve their bone regenerative properties. This has led to the development of a novel xenogeneic particulate bone substitute, with integrated hyaluronate molecules.^85^ Once hydrated, the mixture becomes a sticky bone, thus improving the surgical handling and maintenance of the graft. To our knowledge, no clinical information exists about the advantage of the sticky bone form in comparison to the manual mixture of HA gel to particulate substitute.

Mucogingival surgery focuses on correcting problems related to the gingiva and surrounding soft tissues. Its objective is to enhance smile aesthetics, treat gingival recessions, reconstruct lost tissues, and restore a healthy gingival architecture. Recently, the use of hyaluronic acid in mucogingival surgery has gained popularity as a valuable therapeutic tool for improving the health and aesthetics of the soft tissues in the oral cavity.^86^ Progressively, surgical grade HA has been tested in soft tissue coverage procedures to improve the obtained clinical results in terms of root coverage, connective tissue thickening, and even periodontal attachment regeneration.^87^ In periodontology, hyaluronic acid appears to be a promising tool in these interventions due to its unique properties such as hydrophilic and volumizing properties.

## 6 Conclusion

Research on Hyaluronic acid has shown encouraging results when used in periodontal regenerative procedures. Its enhancing effects on bone and soft tissue cells make it an ideal biological product for surgical soft tissue augmentation and periodontal/pre-implant bone regeneration. Long-term, randomized controlled trials are still needed to assess the real clinical benefit of adding this product to the current surgical protocols.
